# Silencing of lncRNA UCA1 inhibited the pathological progression in PCOS mice through the regulation of PI3K/AKT signaling pathway

**DOI:** 10.1186/s13048-021-00792-2

**Published:** 2021-03-20

**Authors:** Dongyong Yang, Yanqing Wang, Yajing Zheng, Fangfang Dai, Shiyi Liu, Mengqin Yuan, Zhimin Deng, Anyu Bao, Yanxiang Cheng

**Affiliations:** 1 Department of Obstetrics and Gynecology, Renmin Hospital of Wuhan University, Wuhan, China; 2 Department of Clinical Laboratoy, Renmin Hospital of Wuhan University, Wuhan, China

**Keywords:** UCA1, PCOS, Inflammation, lncRNA, Granulosa cell

## Abstract

**Background:**

Polycystic ovary syndrome (PCOS) is the most common hormonal disorder among reproductive-aged women worldwide, however, the mechanisms and progression of PCOS still unclear due to its heterogeneous nature. Using the human granulosa-like tumor cell line (KGN) and PCOS mice model, we explored the function of lncRNA UCA1 in the pathological progression of PCOS.

**Results:**

CCK8 assay and Flow cytometry were used to do the cell cycle, apoptosis and proliferation analysis, the results showed that UCA1 knockdown in KGN cells inhibited cell proliferation by blocking cell cycle progression and promoted cell apoptosis. In the in vivo experiment, the ovary of PCOS mice was injected with lentivirus carrying sh-UCA1, the results showed that knockdown of lncRNA UCA1 attenuated the ovary structural damage, increased the number of granular cells, inhibited serum insulin and testosterone release, and reduced the pro-inflammatory cytokine production. Western blot also revealed that UCA1 knockdown in PCOS mice repressed AKT activation, inhibitor experiment demonstrated that suppression of AKT signaling pathway, inhibited the cell proliferation and promoted apoptosis.

**Conclusions:**

Our study revealed that, in vitro, UCA1 knockdown influenced the apoptosis and proliferation of KGN cells, in vivo, silencing of UCA1 regulated the ovary structural damage, serum insulin release, pro-inflammatory production, and AKT signaling pathway activation, suggesting lncRNA UCA1 plays an important role in the pathological progression of PCOS.

## Background

Polycystic ovary syndrome (PCOS) is a common hormonal disorder among reproductive-aged women worldwide. Women with PCOS often have high levels of male hormones and increased levels of inflammation [1, 2]. Hyperandrogenism, ovulatory dysfunction and polycystic ovaries are typical characteristics of PCOS, which lead to compensatory hyperinsulinemia and anovulatory infertility [3, 4]. Although the study of PCOS began in 1953, the mechanisms and pathogenesis of PCOS remain unclear due to its heterogeneous nature [5]. Studies suggest that PCOS may be associated with the survival and proliferation of granulosa cell (GC), GCs from PCOS patients have a higher proliferation and lower apoptotic rate compared to non-PCOS women [6, 7], inhibition of proliferation ability of GCs may contribute to PCOS therapy [8]. KGN is a type of human granulosa like tumor cell line, which maintains most of the normal GCs physiological characteristics, due to its good growing ability in culture, KGN cells are normally applied to the study of the molecular mechanism and function of GCs [9].

The ENCODE project announced that only 2 % of the genome was made up of coding sequences, which means, a large part of the genome will be transcribed into non-coding RNAs, including small nuclear RNAs (snRNAs), microRNAs, circular RNAs, piwi-interacting RNA, and Long non-coding RNAs (lncRNAs). lncRNA is one popular type of non-coding RNA, which with a length of more than 200 nucleotides, could take part in a variety of disease processes through the regulation of cell cycle and cell differentiation [10]. According to recent studies, genetic abnormalities, especially lncRNAs, play major roles in the development of PCOS [11–13]. It was also found that different lncRNAs could have different functions in the apoptosis and proliferation of human granulosa-like tumor cell line KGN cells. For example, lncRNA BANCR and lncRNA LET could induce the apoptosis of KGN cells by the inhibition of the KGN cell proliferation [9, 14], however, lncRNA SRA was demonstrated to contribute to cell proliferation and suppress the GCs apoptosis [15].

LncRNA urothelial carcinoma‑associated 1 (UCA1) acts as an oncogene in a series of cancer types, such as gastric cancer, bladder cancer and colorectal cancer [16]. UCA1 has three exons that encode a 2.2-kb and 1.4-kb isoform, and the 1.4-kb isoform was first identified as a urine marker in bladder cancer [17]. UCA1 is highly expressed in tumor tissues, regulating the metastasis, invasion, proliferation, and apoptosis of tumor cells [18, 19]. Colorectal cancer patients with a higher UCA1 expression had a poorer prognosis [20]. It is reported that UCA1 can modulate the cell growth and apoptosis of breast cancer by downregulating the expression of microRNA-143 [21]. All these findings suggest an important role of UCA1 in the development of cancer.

However, the role of UCA1 in PCOS is not much studied. In this study, we aimed to find out the role of UCA1 in PCOS. The results showed that UCA1 had a higher expression in PCOS GCs compared to normal. In vitro, we investigated the potential role of UCA1 in cell proliferation and apoptosis using the KGN cell line. In vivo, lentivirus containing sh-UCA1 injected into the ovary of PCOS mice, the function of UCA1 in the pathology of PCOS was evaluated by serum insulin assay and pro-inflammation cytokine secretion. In addition, we investigated whether UCA1 participated in the development of PCOS through the regulation of AKT signaling pathway.

## Materials and Methods

### Animal

Female C57BL/6 mice (25-day old) were obtained from the Guangdong Medical Laboratory Animal Center. All mice were maintained in a 12 h light/12hr dark cycle with enough food and water. All the experiments carried on mice followed the National Institutes of Health guidelines.

60 mg/kg dehydroepiandrosterone (DHEA, GAOYUANbio, China) was injected daily for three weeks to induce PCOS. An equal volume of sesame oil was injected into the control mice at the same time. The PCOS mice were separated into two groups, each group subcapsular injected 5 × 10^8^ PFU/mL lentivirus into the ovary. Lentivirus injected into the sh-NC group, carrying an empty negative control vector, and the lentivirus in the sh-UCA1 group carrying a lncRNA UCA1 specific shRNA. Insulin release assay was performed after 2 weeks of lentivirus infection, and the ovaries were collected for histological examination, real-time PCR, ELISA, TUNEL and Western blot, six mice were selected for each experiment per group.

### Primary GCs extraction and cell line culture

Follicles picked out from mice ovaries were washed in PBS, cut into pieces and digested with 0.1 % collagenase type I (Thermo Fisher Scientific, USA) for 5 min at room temperature. Centrifuged the suspension at 300 g for 10 min and washed by PBS three times, the GCs were collected.

The human GC tumor-derived cell line, KGN, was purchased from CELLCOOK biotechnology. Cells were cultured in nutrient mixture F-12 Ham /Dulbecco’s modified Eagle medium contained 10 % fetal bovine serum at 37℃, with 5 % CO_2_. Cells were transfected with sh-NC or sh-UCA1, 48 h after transfection, the cells were collected for the following experiments. For inhibitor experiment, KGN cells were treated with 50 μm PI3K inhibitor LY294002 (Cell Signaling Technology, Inc.) for 24 h.

### qRT-PCR

Total RNA from KGN cells was isolated using TRIzol reagent (Invitrogen, USA) according to its manufacturer’s protocol. MultiscribeRTkit (Biosystems, Spain) was used for RNA reverse transcription. qRT-PCR was performed by SYBR Green real-time PCR Master Mix (Toyobo, Osaka, Japan). The primer sequences were designed as previously described [11]. GAPDH serves as an internal control. The relative expression of mRNAs and lncRNA was calculated using the 2^−ΔΔCt^ method.

### CCK-8 assay

Cells were seeded into 96-well plates with 3*10^3^ cells per well until cell attachment. After transfection, cells were cultured to each indicated time, 10 µL Cell Counting Kit-8 solutions (CCK-8, yeasen, china) was added to each well and incubated for 2 h. The 450 nm wavelength optical density (OD) was measured by a microplate reader (BIOTEK, Vermont, USA).

### Flow cytometry for apoptosis and cell cycle analysis

For apoptosis analysis, Annexin V-FITC / PI Apoptosis Detection Kit (Elabscience Biotechnology Co., Ltd, China) was used to stain cells. Different parts in the analysis image represented different cell states, the upper right part represented cells at the late-apoptotic stage, the lower right part represented the early apoptotic cells, and the lower-left part represented viable cells. To analyze cell cycle distribution, cells were stored at -20 ℃ after fixed in pre-cold 70 % ethanol for 20 min. Then, cells were stained with RNase and PI reagent from Cell Cycle Analysis Kit (C1052, Beyotime) according to the standard guide. After incubation, cells were analyzed by the FACSCaliber flow cytometer.

### Hematoxylin-eosin (H&E) staining

The ovarian tissue were fixed in 10 % formalin for 24 h, then, embedded in paraffin and cut into 5 μm thick sections. The staining was performed using H&E Staining Kit (Abcam, USA) following its standard tutorial. Sections were covered by Hematoxylin, Mayer’s (Lillie’s Modification) and incubate for 5 min, then washed by distilled water twice. Applied Bluing Reagent to cover tissue section and incubate for 15 s. Wash and dip slide in absolute alcohol and blot excess off. Covered sections with Eosin Y Solution (Modified Alcoholic) and incubate for 3 min. Dehydrated, Cleared, and mounted slide in synthetic resin. The granulosa cells were counted per follicle. The average values of five follicles in a section and three sections from an ovary were analyzed.

### Serum insulin and testosterone assay

After 12 h-fasting, mice were given 2 g/Kg glucose by gavage. Before gavage and 30, 60, 90, 120 min after gavage, retro-orbital sinus puncture was used to collect blood samples from medial canthus of the eye. The serum insulin was assayed using Insulin Mouse ELISA Kit (Invitrogen, USA). The area under the curves (AUC) was calculated. The serum testosterone was assayed using Testosterone ELISA Kit (Abcam, USA).

### ELISA

ELISA kits bought from Sino Biological (Beijing, china) was used for the measurement of the contents of tumor necrosis factor -α (TNF-α), IL-6 and interleukin (IL)-1β. The coefficient of variation in ELISA batch was 3.5 %.

### Terminal deoxynucleotidyl-transferase mediated dUTP nick-end labeling (TUNEL) assay

Firstly, GCs collected from mice model were fixed with paraformaldehyde (4 %) for 1 h and permeabilized with 0.1 % Triton-X100 for 2 min. ApopTag peroxidase in situ apoptosis detection kit (Oncor, USA) was used to perform the experiment according to the manual. Then the cells were counterstained with DAPI (2 µg/ml, 4′,6-diamidino-2-phenylindole, Sigma-Aldrich, USA) in dark for 5 min at room temperature. Three random selected fields in each sample were observed, and the ratio of positive apoptotic cells/total number of nuclei was calculated.

### Western blot

Ovarian tissues were lysed by cooled RIPA Lysis and Extraction Buffer (Thermo Fisher Scientific, USA). After 10,000 × g centrifugation at 4 ℃ for 10 min, Nuclear Protein Extraction Isolation (Fractionation-Translocation) Kit (Fivephoton Biochemicals, USA) was used to isolated nuclear protein. After separated by SDS-PAGE gels, protein samples were transferred onto PVDF membranes. After 5 % no-fat milk blocked the membranes at room temperature. The membranes were incubated with anti‑phospho(p)‑AKT (1:2,000, #ab8933, Abcam), Anti‑AKT (1:1,000, #ab8805, Abcam) and anti‑GAPDH (1:2,000, #ab181603, AbMart Bio‑tech Co. Ltd.,) overnight at 4℃. Subsequently, the membranes were incubated with secondary antibodies for 1 h at room temperature.

### Statistical analysis

SPSS and GraphPad were used to do the analysis. The results were presented as mean ± standard deviation (SD). statistical significance among groups was analyzed by the one‑way analysis or Student’s t‑test analysis. A p-value less than 0.05 was considered statistically significant.

## Results

### Silencing of UCA1 regulated the proliferation and apoptosis of KGN cells

**Fig. 1:**
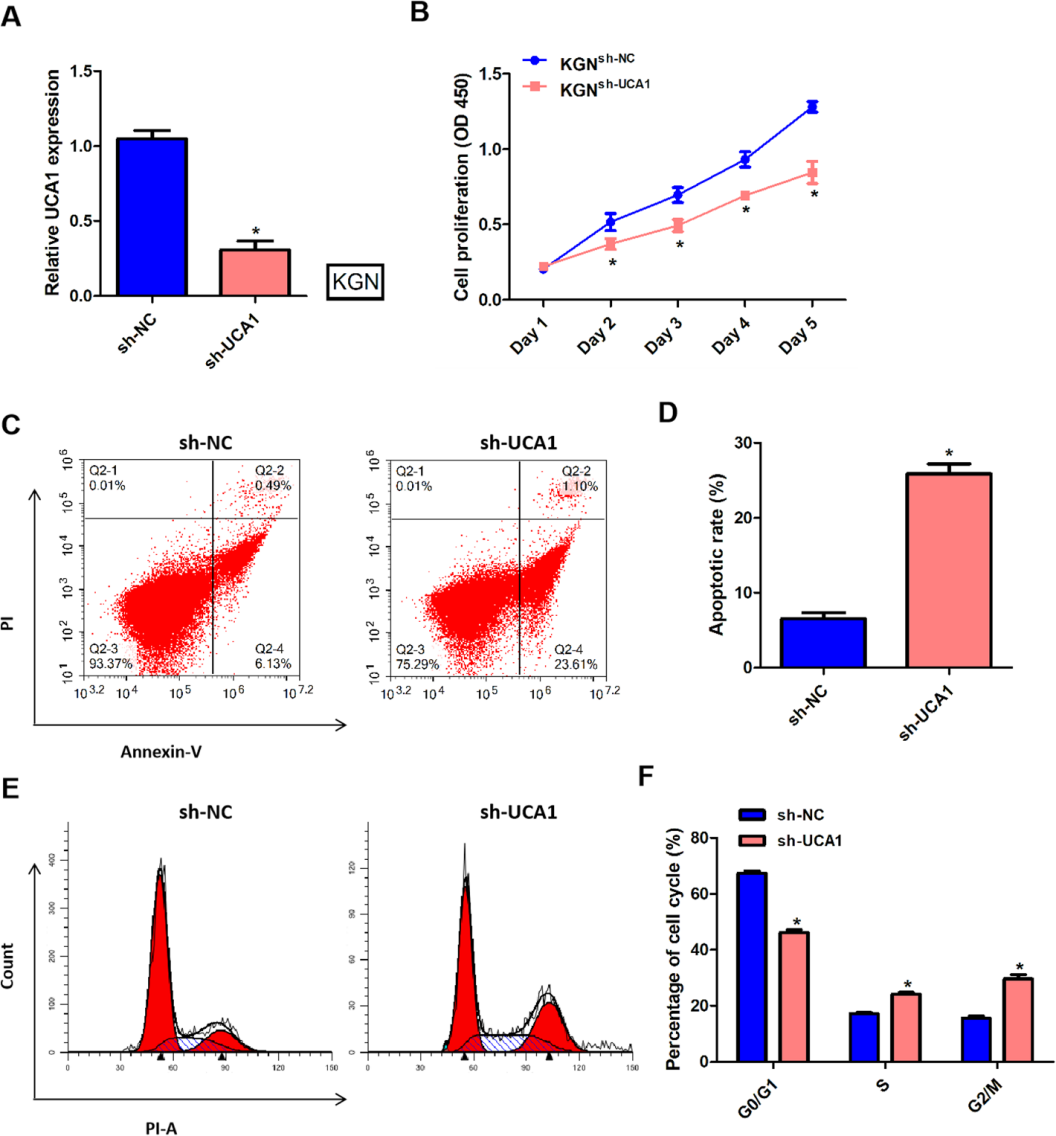
Silencing of UCA1 regulated the proliferation and apoptosis of KGN cells. The sh-UCA1 or sh-NC was transfected into KGN cells to knockdown the expression of UCA1. The cells were cultured for 48 h after transfection. **a** qPCR showed that the expression of UCA1 in KGN cells was downregulated, after transfected with sh-UCA1. **b**: The cell proliferation was detected by CCK-8 assay per day (day 1 to day 5). **c**-**d** Annexin-V/PI staining revealed cell apoptosis rate of KGN cells transfected with sh-UCA1 increased. **e**-**f** Flow cytometer analysis detected cell cycle distribution in KGN cells. *p < 0.05 showed significant difference.

To determine the roles of UCA1 in the proliferation and apoptosis of KGN cells, we transfected sh-UCA1 and sh-NC into KGN cells to knock down the expression of UCA1. After transfection with sh-UCA1, the expression of UCA1 in KGN cells was significantly decreased (Fig. 1 a). Using CCK8-assay to detect cell proliferation rate for 5 days, the results showed that silencing of UCA1 in KGN cells prominently suppressed the cell proliferation (Fig. 1b). Figure 1 c and d examined the effects of UCA1 on apoptosis of KGN cells. The results showed that downregulated of UCA1 significantly increased the apoptotic rate of KGN cells. Results from Fig. 1e and f displayed knockdown of UCA1 induced cell cycle progression of KGN cells from G0/G1 phase to S/G2/M phase. All these results indicated that silencing of UCA1 suppressed the proliferation and promoted the apoptosis of KGN cells.

### Silencing of UCA1 attenuated the pathological characteristics of PCOS mice

**Fig. 2:**
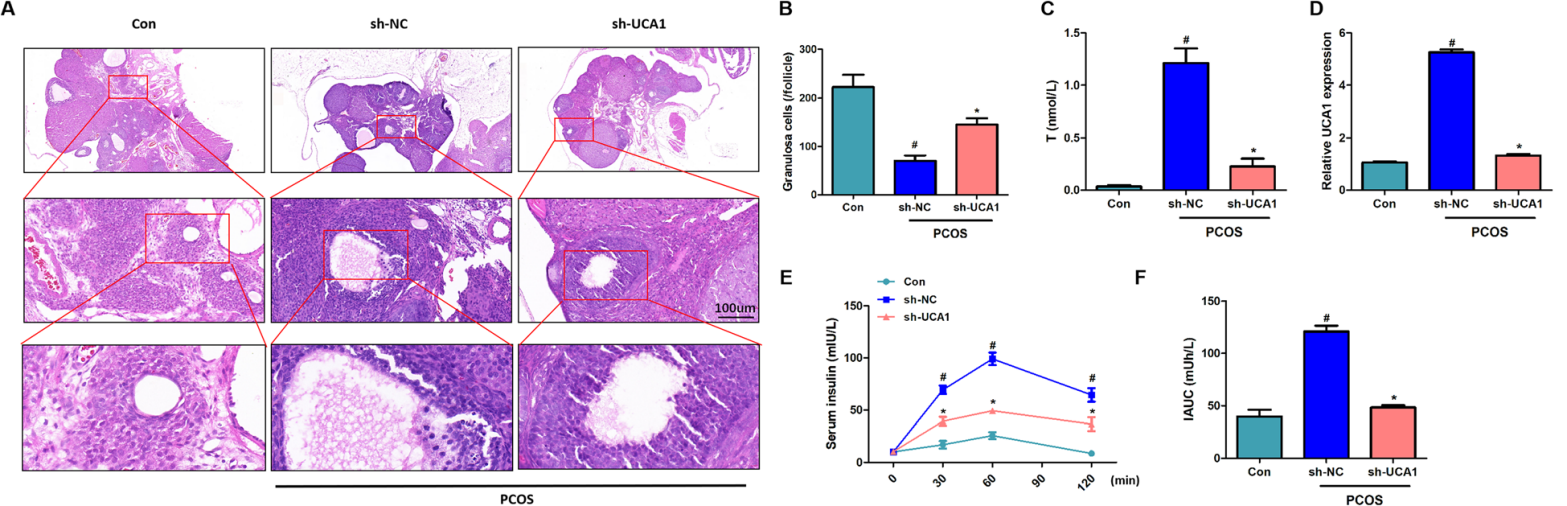
Silencing of UCA1 attenuated the pathological characteristics of PCOS mice. Lentivirus containing sh-UCA1 or sh-NC was infected into the ovary of the PCOS mice, control mice infected with lentivirus carrying an empty negative control vector. **a** H&E staining result of the ovary. Scale bar: 100 μm. **b** Number of granular cells per follicle. **c** The serum testosterone level was downregulated by UCA1 silencing. **d** The expression level of UCA1 in normal (Con) and PCOS mice. LncRNA UCA1 expression was enhanced in PCOS mice and decreased after sh-UCA1 treatment. **e** The release level of serum insulin was increased in PCOS mice and sh-UCA1 downregulated the release. **f** Area under the curve (IAHC) of serum insulin level. Data were mean ± SD (*n* = 6), #*p* < 0.05 showed significant difference vs. control, *p < 0.05 showed significant difference vs. PCOS-sh-NC.

The H&E staining showed that, compared to the control ovarian tissue, PCOS mice had a disorder arranged structure of ovarian tissue, and represented granular cell layer thinning, ovarian cystic expansion, granular cells decreasing. Silencing of UCA1 attenuated the ovary structural damage, thickened the granule cell layers, and increased the number of granular cells (Fig. 2 a-b).

Lentivirus containing UCA1-specific shRNA was injected into the ovary of the PCOS mice and significantly downregulated the lncRNA UCA1 expression (Fig. 2d), additionally, the serum testosterone was reduced by UCA1 silencing (Fig. 2 c). Through serum insulin assay, we tested the insulin release levels in mice per day for 5 days, the results showed that PCOS mice showed higher insulin release compared to control and silencing of UCA1 could significant reduced insulin release (Fig. 2e). The IAUC quantified the change levels of serum insulin (Fig. 2 f), the IAUC increased in the PCOS-sh-NC group and decreased after silencing of UCA1.

### Silencing of UCA1 inhibited the production of pro‐inflammatory cytokines

**Fig. 3:**
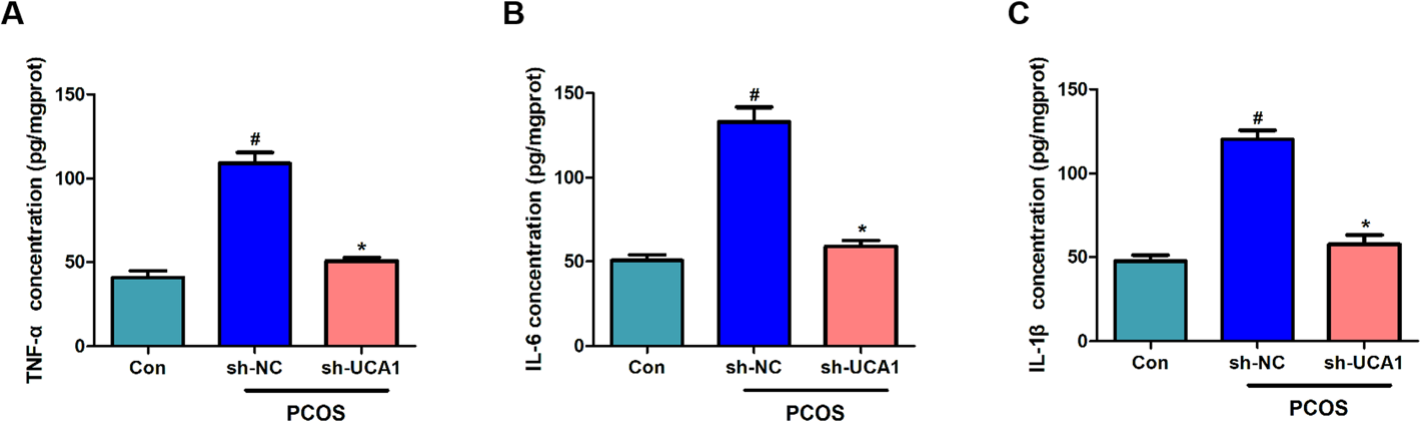
Silencing of UCA1 inhibited the production of pro-inflammatory cytokines. ELISA detected the concentration of TNF-α (**a**), IL-6 (**b**) and IL-1β (**c**) in the ovary. Silencing of lncRNA UCA1 downregulated the release of pro-inflammatory cytokines in the ovary of PCOS mice. Data were mean ± SD (*n* = 6). #*p* < 0.05 showed significant difference vs. control, *p < 0.05 showed significant difference vs. PCOS-sh-NC.

**Fig. 4:**
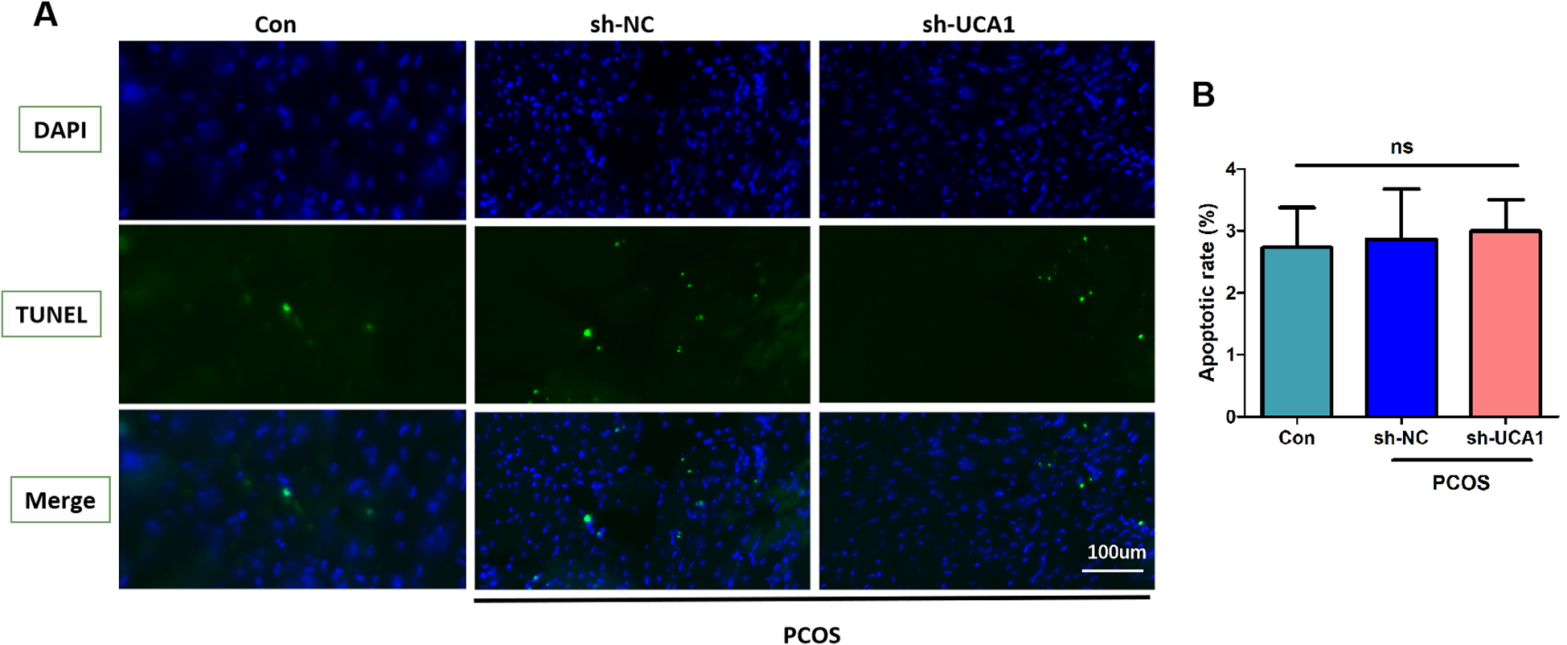
The apoptosis of GCs in vivo was not affected by UCA1. **a** Representative image of TUNEL staining (*n* = 6, scale = 100 μm). **b** The apoptotic rate of GC cells (*n* = 6). ns means no statistical difference.

Using ELISA, we found that the levels of typical pro-inflammatory cytokines (including TNF - α, IL-6, and IL-1 β) in the ovarian tissues of PCOS mice were increased significantly. In addition, UCA1 knockdown predominantly reduced the production of pro-inflammatory cytokines in PCOS mice (Fig. 3 a-c). The results suggested that silencing of UCA1 inhibited PCOS inflammation. The apoptotic of GCs was measured by TUNEL analysis, the results showed that GCs apoptosis was not affected by UCA1 (Fig. 4 a-b).

### Silencing of UCA1 regulated cell proliferation and apoptosis through the repression of AKT signaling pathway

**Fig. 5:**
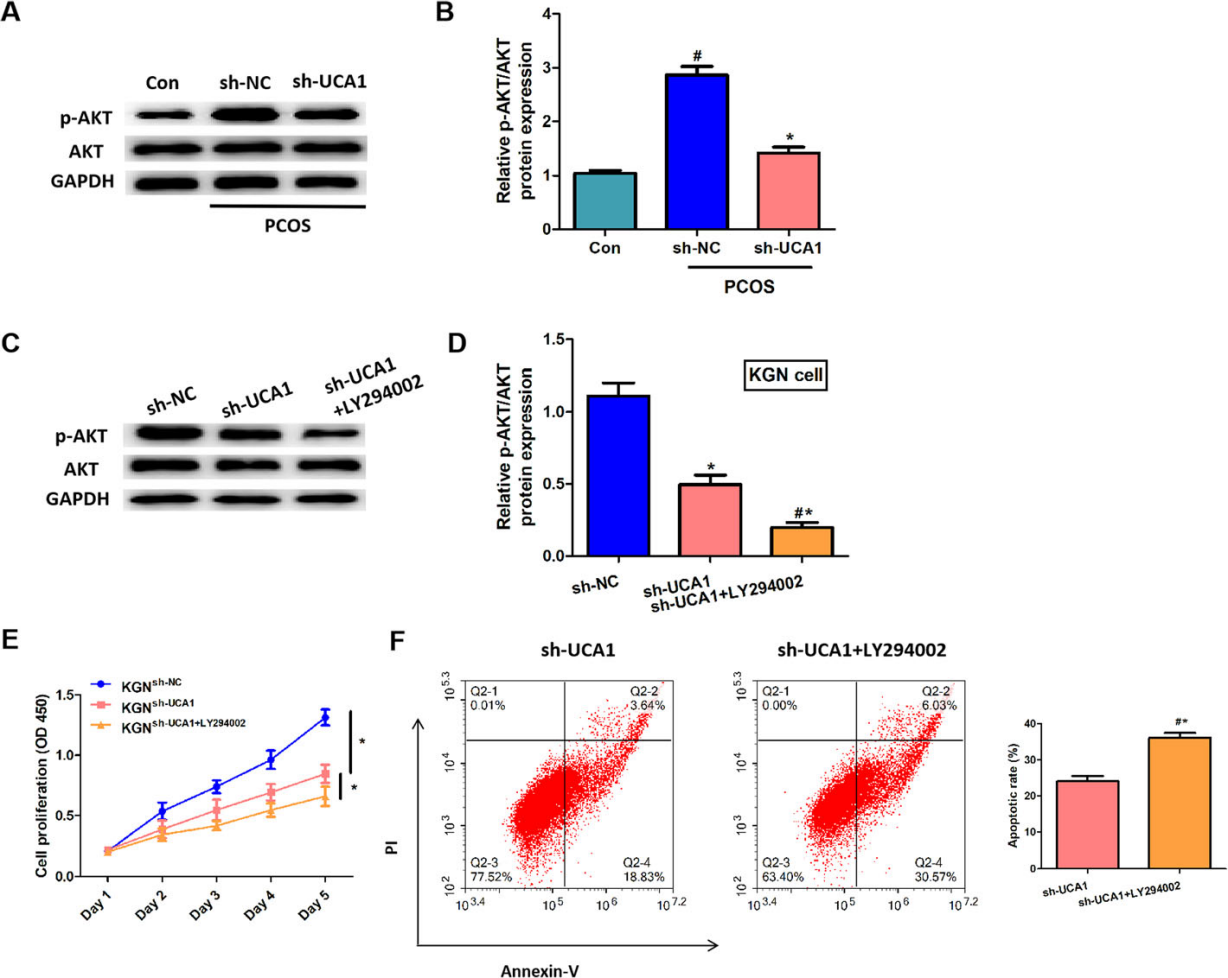
Silencing of UCA1 downregulated the expression of p-AKT/AKT both in vivo and in vitro. **a**: Western blot detected the protein expression level of p-AKT and AKT in mice ovaries. **b**: The relative expression level of p-AKT/AKT, normalized to GAPDH. **c**: Western blot detected the protein expression level of p-AKT and AKT in KGN cells. **d**: The relative expression level of p-AKT/AKT, normalized to GAPDH. **e**: The cell proliferation was detected by CCK-8 assay per day (day 1 to day 5). **f**: Apoptosis rate of KGN cells. Data were mean ± SD (*n* = 6). ^#^*p* < 0.05 showed significant difference vs. control, *p < 0.05 showed significant difference vs. PCOS-sh-NC. ^#^*p < 0.05 showed significant difference vs. PCOS-sh-UCA1.

AKT signaling pathway has a vital role in cell cycle distribution, cell growth,apoptosis and survival of human cancer [22], and UCA1 could regulate cell cycle progression in breast cancer via PI3K/AKT-dependent pathway [23]. Here, we explored whether UCA1 could regulated AKT pathway in PCOS. Western blot showed that the relative density of p-AKT/AKT was significantly increased in the ovary of PCOS mice and reduced by UCA1 knockdown (Fig. 5 a-d). The results suggested that silencing of UCA1 suppressed the AKT activation. In our inhibition experiment, we validated the suppression role of LY294002 in p-AKT/AKT expression (Fig. 5 c-d), LY294002 inhibited the proliferation and promoted the apoptosis of KGN cells (Fig. 5e-f), which indicated that AKT signaling pathway regulated the proliferation and apoptosis of KGN cells.

## Discussion

The exact cause of PCOS is still a mystery. Excess insulin and low-grade inflammation might be 2 main factors that contribute to PCOS development. In the past decades, researchers commonly believed that PCOS is caused by genetic abnormalities, and their focus is mainly on mRNA transcriptome. However, more and more evidence has proved that epigenetic regulation and non-coding RNA are involved in the regulation of PCOS development. Pooja Sagvekar et al., found that 2509 CpGs representing 1777 genes showed hypermethylation while 2977 within 2063 genes showed hypomethylation in PCOS. These differential methylation sites in PCOS were associated with chemokine/cytokine-mediated inflammation, angiogenesis, endothelin/integrin signaling, which may contribute to ovarian defects by regulating follicular development [24]. In the chronically androgenized rat PCOS model, there are 24 % of 349 miRNAs are differentially expressed compared to normal rat. These differentiated miRNAs mainly localized in the follicles could regulate ovarian pathology by participating in post-transcriptional regulation of genes [25]. Additionally, more lncRNA are demonstrated to play an important role in PCOS. Yu-Dong Liu et al., compared the expression profiles of lncRNA in GCs between PCOS patients and healthy women. They found that 862 lncRNA transcripts were differentially expressed in the GCs of PCOS patients compared with those from healthy individuals, which suggested that lncRNAs dysregulation could participate in the pathogenesis of PCOS [11]. In the cumulus cells of PCOS patients, the lncRNA PWRN2 expression was increased, PWRN2 was demonstrated to contribute to the maturation of oocyte nuclear in PCOS by acting as a ceRNA to reduce the binding ability of miR-92b-3p for TMEM120B [26].

lncRNA UCA1 is often dysregulated in cancer and could act as an oncogene promoting cell proliferation and migration in many different types of human cancers. In gastric cancer, UCA1 was aberrantly increased and promoted tumor metastasis by significantly induced ZEB2 expression through sponging miR-203 [27]. In colorectal cancer, UCA1 was upregulated in the patients’ serum exosomes and increased the ability of proliferation and migration in cancer cells [28]. However, the function of UCA1 in PCOS is unknown. In this study, we used KGN cells to infer the ability of UCA1 to GC cell proliferation and apoptosis. Knocking down the expression of UCA1 in KGN cells inhibited cell proliferation and promoted cell apoptosis, the result was confirmed by G_2_/M phase cell cycle arrested in the sh-UCA1 KGN cell group, which indicated that UCA1 might have a potential role in PCOS progression through the regulation of GCs. However, in the in vivo experiments, H&E staining showed that silencing of UCA1 increased GCs, the TUNEL staining showed that the apoptosis of GCs in PCOS mice was not affected by UCA1, which was not consistent with the in vitro results. Considering the complexity of the internal environment and the mechanism of GCs proliferation in PCOS is not well explored, there are a number of factors that can interfere with the effect of UCA1. According to previous research, high insulin concentration could regulate AKT/mTOR pathway and increase the proportion of apoptosis/proliferation [29], which was matched with our H&E data. In vivo, UCA1 could downregulate the insulin release, so we suspected that there has a competitive regulation mechanism between UCA1 and insulin in the regulation of GCs proliferation, which may cause controversial results, due to the complicated internal environment, the actual reason needs further investigated.

Furthermore, to find out how UCA1 regulated the pathological progression of PCOS, we constructed a PCOS mouse model by injected female C57BL/6 mice with lentivirus carrying lncRNA UCA1 specific shRNA. The H&E staining results validated the construction of the PCOS mice model, silencing of UCA1 attenuated the ovary structural damage, thickened the granule cell layers, increased the number of granular cells, which rescued the pathological character of PCOS. The high release level of serum testosterone and insulin were two main significant characters of PCOS. In the present researches, insulin resistance has been demonstrated to contribute to the development of PCOS [30], insulin resistance and hyperinsulinemia directly affected the functions of ovaries [31]. Yan Li et al., found that serum insulin level was significantly increased in PCOS mice, which may refer to hyperinsulinemia [32]. In vitro experiments demonstrated that the productions of androstenedione, progesterone, and testosterone can be prominently increased by insulin receptors in cultured polycystic ovary theca cells [33]. Our insulin release assay showed that the serum insulin and testosterone level was significantly increased in PCOS mice and both reduced by silencing of UCA1. According to previous study, UCA1 could regulate testosterone level through insulin release, but how UCA1 regulated insulin release has never ever been discussed. The mechanism of the regulation of UCA1 to insulin need further investigate. As insulin resistance has been reported to be related to inflammation [1], high-level insulin release promotes the production of angiogenic factors and pro-inflammatory cytokines, and glucose uptake was decreased in the inflammatory environment, which may aggravate insulin resistance [34]. Chronic low-grade inflammation has been demonstrated to involve in the development of PCOS [35]. We measured the concentration of three pro-inflammatory cytokines TNF-α, IL-6, and IL-1β in the ovary of PCOS mice. All these cytokines were demonstrated to have a higher expression in PCOS patients than normal individuals. The dysregulation of these pro-inflammatory cytokines could delay follicular maturation and induce altered steroidogenesis [36]. In our data, PCOS mice had a significantly increased production of the pro-inflammatory cytokine, which was consistent with the previous study, and sh-UCA1 markedly inhibited the production of pro-inflammatory cytokine. Combined with previous reports, a high level of insulin promoted the initiation of inflammation, and insulin resistance was aggravated by the inflammatory environment [34, 37]. Our data indicated that lncRNA UCA1 participated in the pathophysiology of PCOS, through an insulin-inflammation cycle.

The PI3K/AKT pathway is commonly involved in cellular proliferation and survival, and is considered to plays a vital role in the normal cell cycle progression. AKT is one of the key molecular of PI3K/AKT pathway, also known to contribute to metabolism and angiogenesis. According to previous studies, UCA1 participated in many disease progressions through the regulation of PI3K/AKT signaling pathway [38, 39]. In breast cancer, UCA1 expression was positively correlated with AKT expression, the activation of PI3K/AKT pathway can be regulated by UCA1 to confer tamoxifen resistance in cancer [12]. At present, some reports have mentioned that some molecules regulated PCOS progresses through PI3K/AKT signaling activation. For example, growth hormone and plumbagin activated the PI3K/AKT signaling pathway and alleviated cell apoptosis in PCOS [40, 41]. Here, we investigated whether UCA1 regulated PI3K/AKT signaling pathway in PCOS mice. The expression level of pAKT/AKT in PCOS mice is prominent high, compared to the control group, silencing of UCA1 suppressed the AKT signaling pathway. Using LY294002 to inhibit AKT signaling activation, the proliferation and apoptosis of KGN cells were influenced. Taken together, we indicated that UCA1 regulated the characters of PCOS through AKT signaling pathway. However, the function of AKT signaling in PCOS mice needs further validation.

## Conclusions

In conclusion, we firstly demonstrate that UCA1 plays an important role in PCOS mice. Silencing of UCA1 inhibited most pathological progression in PCOS, including GC proliferation, ovary structural damage, serum insulin release and pro-inflammation production, through the suppression of AKT signaling pathway activation. Our study innovatively described the role of UCA1 in PCOS mice, we speculated that UCA1 could serve as a new target for the therapy of PCOS, provided enlightenment for the research related to PCOS.

## Data Availability

The datasets used and/or analysed during the current study are available from the corresponding author on reasonable request.
